# Is ^123^I-metaiodobenzylguanidine heart-to-mediastinum ratio dependent on age? From Japanese Society of Nuclear Medicine normal database

**DOI:** 10.1007/s12149-018-1231-6

**Published:** 2018-01-15

**Authors:** Kenichi Nakajima, Koichi Okuda, Shinro Matsuo, Hiroshi Wakabayashi, Seigo Kinuya

**Affiliations:** 1 Department of Nuclear Medicine, Kanazawa University Hospital, 13-1 Takara-machi, Kanazawa, 920-8641 Japan; 2 Department of Physics, Kanazawa Medical University, Uchinada, Kahoku, Japan

**Keywords:** Scintigraphy, Sympathetic imaging, Quantitation, Aging, Standardization

## Abstract

**Background:**

Heart-to-mediastinum ratios (HMRs) of ^123^I-metaiodobenzylguanidine (MIBG) have usually been applied to prognostic evaluations of heart failure and Lewy body disease. However, whether these ratios depend on patient age has not yet been clarified using normal databases.

**Methods:**

We analyzed 62 patients (average age 57 ± 19 years, male 45%) derived from a normal database of the Japanese Society of Nuclear Medicine working group. The HMR was calculated from early (15 min) and delayed (3–4 h) anterior planar ^123^I-MIBG images. All HMRs were standardized to medium-energy general purpose (MEGP) collimator equivalent conditions using conversion coefficients for the collimator types. Washout rates (WR) were also calculated, and we analyzed whether early and late HMR, and WR are associated with age.

**Results:**

Before standardization of HMR to MEGP collimator conditions, HMR and age did not significantly correlate. However, late HMR significantly correlated with age after standardization: late HMR = − 0.0071 × age + 3.69 (*r*^2^ = 0.078, *p* = 0.028), indicating that a 14-year increase in age corresponded to a decrease in HMR of 0.1. Whereas the lower limit (2.5% quantile) of late HMR was 2.3 for all patients, it was 2.5 and 2.0 for those aged ≤ 63 and > 63 years, respectively. Early HMR tended to be lower in subjects with the higher age (*p* = 0.076), whereas WR was not affected by age.

**Conclusion:**

While late HMR was slightly decreased in elderly patients, the lower limit of 2.2–2.3 can still be used to determine both early and late HMR.

## Introduction

The prognoses of patients with heart failure (HF) and Lewy body diseases including dementia with Lewy body (DLB) and Parkinson disease have been predicted based on the uptake of ^123^I-metaiodobenzylguanidine (MIBG). Decreased heart-to-mediastinum ratio (HMR), such as 1.6, 1.68, and 1.74 are predictors of serious cardiac events and cardiac death among patients with HF [1–5]. Reports indicate that decreased HMR (< 1.74 and 2.2) is one variable for differentiating Alzheimer disease (AD) from Lewy body diseases [6–9]. Although some threshold values have been proposed for diagnostic purposes, the influence of age on ^123^I-MIBG parameters has not been clarified. While age is a significant predictor of cardiac events [3, 5, 10, 11] due to higher comorbidities such as diabetes, hypertension, and other coronary risk factors, the impact of age on HMR in patients with a very low likelihood of cardiac disease has not been established. Furthermore, the prognosis of patients with HF has been estimated using washout rates (WR), which also require standardization [12–14].

The present study aimed to determine the effect of age on HMR and WR using the Japanese Society of Nuclear Medicine (JSNM) working group normal database [15, 16], in which potential clinical causes of decreased ^123^I-MIBG uptake are minimized.

## Methods

### JSNM working group database

The JSNM working group database for ^123^I-MIBG included patients with a low likelihood of cardiac disease in whom cardiac MIBG study was indicated as well as routine cardiac examinations [16]. The exclusion criteria comprised patients with electrocardiographic evidence of myocardial ischemia, baseline cardiac diseases including coronary artery disease, valvular heart disease and severe arrhythmia, a history of HF, severe liver dysfunction, renal dysfunction, hypertension, diabetes and dyslipidemia managed with medications. Patients who had coronary stenosis of < 50% and those who had no indications for coronary angiography could be included if they had none of the exclusion criteria listed above. Patients with neurological disorders were also excluded.

The databases (average age, 57 ± 19 years; median age, 63 years; range 20–84 years) contained 37 patients who were assessed using low-energy (LE) collimators (50 ± 19 years, 16 males) and 25 who were assessed using low-medium-energy (LME) or medium-energy (ME) collimator (68 ± 13 years, 12 males) [17]. The HMR determined using LE collimator and a calibration phantom was corrected based on multicenter phantom experiments [18, 19].

### ^123^I-MIBG imaging

Early and late anterior planar images were acquired in 256 × 256 matrices at 15 and at 180–240 min after an intravenous injection of 111 MBq of ^123^I-MIBG. The acquisition time was 180–300 s. The energy for ^123^I was centered at 159 keV with a window of 20%. Early and late planar anterior images were assessed in this study. Early and late HMR (HMR_E_ and HMR_L_) were calculated from circular and rectangular regions of interest (ROI) set on images of the heart and mediastinum, respectively, using a semi-automated ROI setting software [20].

### Conversion of institutional HMR to standardized HMR

According to published phantom-based findings, average conversion coefficients (CC) of various collimators were used to calculate standardized HMR. That is

$${\text{HM}}{{\text{R}}_{{\text{std}}}}=\left( {0.{\text{88}}/{K_{\text{i}}}} \right) \times \left( {{\text{HM}}{{\text{R}}_{\text{i}}} - {\text{1}}} \right)+{\text{1}},$$

where 0.88 is a CC of ME general purpose collimator, *K*_i_ is the CC of the institutional camera-collimator, and HMR_i_ is the institutional HMR.

### Washout rate

Washout rates were calculated from early and late heart counts (*H*_E_ and *H*_L_) and mediastinal counts (*M*_E_ and *M*_L_) using the following formulae for WR_BDC,_ WR_DC and_ WR_HMR_:

WR_BDC_, with background (mediastinal counts) and time-decay corrections:

$$\left( {\left( {{H_{\text{E}}} - {M_{\text{E}}}} \right) - \left( {{H_{\text{L}}} - {M_{\text{L}}}} \right)/{\text{DCF}}} \right)/\left( {{H_{\text{E}}} - {M_{\text{E}}}} \right) \times {\text{1}}00\left( \% \right),$$

where DCF is a decay correction factor calculated as 0.5^(time [h] between early and late images/13).

WR_DC_, decay correction for late image:

$$\left( {{H_{\text{E}}} - {H_{\text{L}}}/{\text{DCF}}} \right)/{H_{\text{E}}} \times {\text{1}}00\left( \% \right).$$

WR_HMR_, WR was calculated from early and late HMR values after standardization of collimators:

$$({\text{HM}}{{\text{R}}_{\text{E}}} - {\text{HM}}{{\text{R}}_{\text{L}}})/{\text{HM}}{{\text{R}}_{\text{E}}}~ \times ~{\text{1}}00\left( \% \right).$$

### Statistics

Data are expressed as means and standard deviation (SD). Goodness-of-fit to the Normal distribution was examined for distribution of HMR using the hypothesis that the data are from Normal distribution (small p values reject the hypothesis by Shapiro–Wilk test). Relationships between ^123^I-MIBG parameters and age were calculated using linear regression analysis. Regression lines and confidence intervals [CI] are shown when values were significant at *p* < 0.10. Differences in variables between groups were determined using *T* tests and analyses of variance. Since the median age was 63 years, the patients were divided into groups according to age ≥ 63 (*n* = 31) and < 63 (*n* = 31) years, respectively. *P* < 0.05 was considered significant.

### Results

Before standardization, distributions of HMR_E_ and HMR_L_ were not Normal distribution (*p* = 0.014 and 0.0003, respectively), whereas after the standardization both distributions of HMR_E_ and HMR_L_ became Normal distributions (*p* = 0.99 and 0.84, respectively) by goodness-of-fit test. Table 1 summarizes the normal values determined from the JSNM working group databases. The normal values were 3.10 ± 0.43 and 3.29 ± 0.48 for HMR_E_ and HMR_L_, respectively. The lower limits (2.5% quantile) of HMR_E_ and HMR_L_ were 2.18 and 2.26, respectively. The mean clearance from the heart or washout calculated using early and late HMR was − 6.5%, indicating that mean HMR_L_ was higher than mean HMR_E_. In fact, HMR_L_ was higher than HMR_E_ in 50 (81%) of 62 of the patients.

**Table 1:** Normal standardized heart-to-mediastinum ratios and washout rates

	Standardized HMR (early)	Standardized HMR (late)	WR, % (background and decay correction)	WR, % (decay correction)	WR, % (calculated using standardized HMR)
Mean	3.10	3.29	13.0	16.0	− 6.5
SD	0.43	0.48	8.4	5.8	8.8
Quantile 2.5%	2.18	2.26	− 3.5	5.9	− 30.2
Quantile 97.5%	3.97	4.39	34.0	30.1	14.2

*SD* standard deviation, *HMR* heart-to-mediastinum ratio

**Fig. 1:**
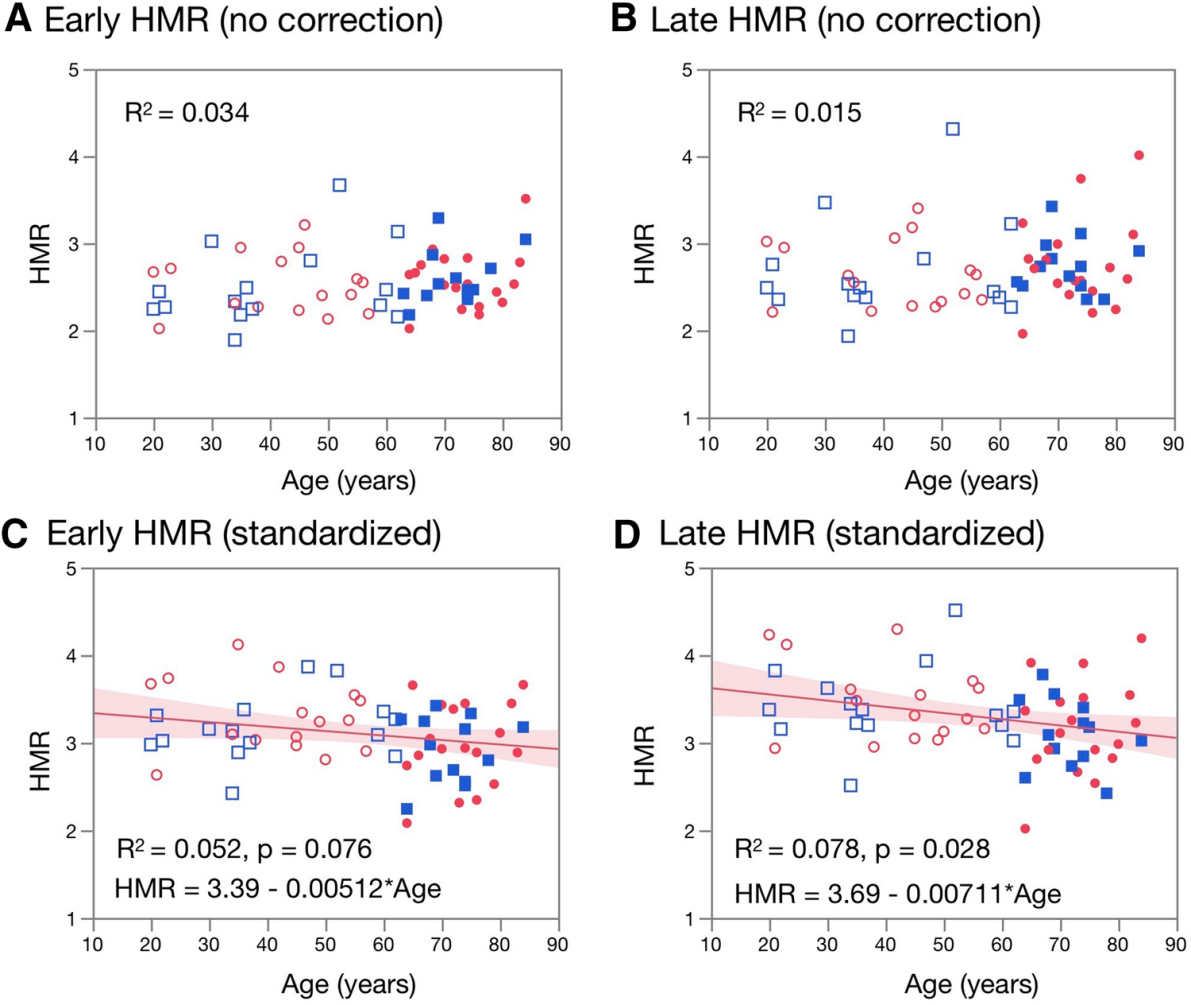
Relationships between age and early and late HMR. **a, b** Early and late HMR before correction of collimator types. **c, d** Early and late HMR after standardization to medium-energy general purpose collimator conditions. Red and blue symbols, female and male individuals, respectively. Unfilled and filled symbols, younger (age < 63 years) and older (age ≥ 63 years) individuals, respectively

The HMR_E_ and HMR_L_ did not significantly correlate with age before standardization of collimators. However, a weakly positive correlation emerged after phantom-based correction to the standardized MEGP condition was applied (Fig. 1) for HMR_L_ (*p* = 0.028), whereas the p value was marginal for HMR_E_ (*p* = 0.076).

**Fig. 2:**
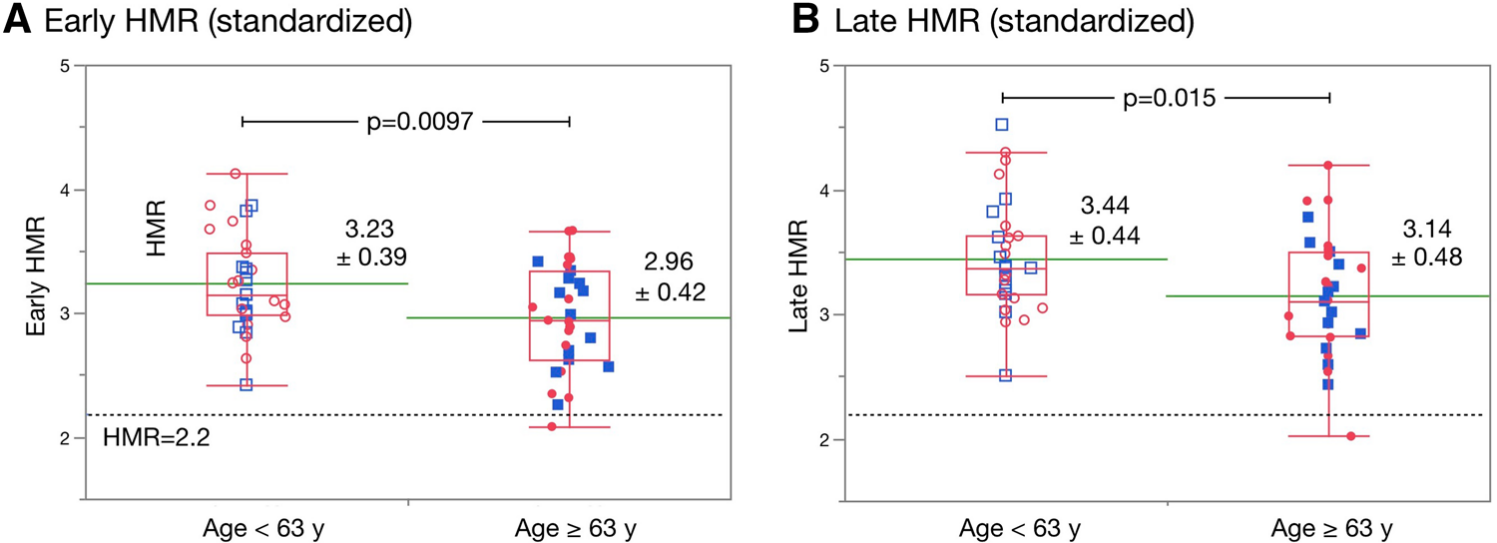
Early and late HMR in younger and older individuals. Early (**a**) and late (**b**) HMR in younger (age < 63 years) and older (≥ 63 years) individuals. Red and blue symbols, female and male individuals, respectively. Unfilled and filled symbols, younger (age < 63 years) and older (age ≥ 63 years) individuals, respectively. Box plot indicates median, 25 and 75% quartiles with whiskers at both ends. Green lines, mean values. Dotted line, HMR = 2.2 (lower limit of normal) as used in Japan using the JSNM working group database [15]

**Fig. 3:**
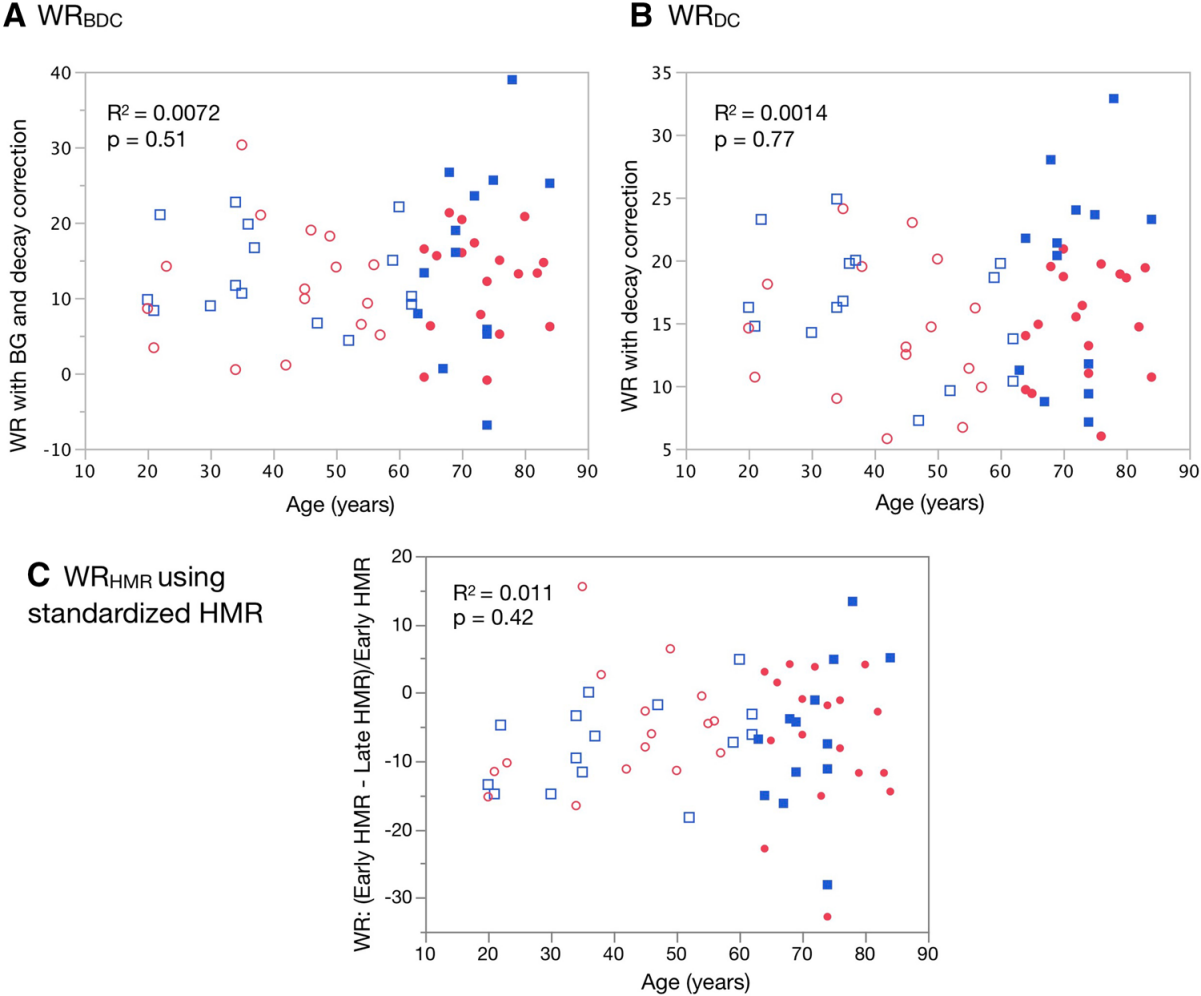
Relationship between age and washout rate (WR). **a** WR_BDC_, WR with background (BG) and decay correction. **b** WR_DC_, WR with decay correction. **c** WR_HMR_, WR calculated using early (HMR_E_) and late HMR (HMR_L_), (HMR_E_-HMR_L_)/HMR_E_

When patients were divided into groups based on a median age (63 years), mean ages of the two groups were 41 ± 14 years and 73 ± 6 years. Goodness-of-fit test showed that HLR_E_ and HMR_L_ were Normal distribution for the two groups (for age < 63 year, *p* = 0.63 and 0.18, respectively; for age ≥ 63 year, *p* = 0.60 and 1.00, respectively). Both HMR_E_ and HMR_L_ were lower in patients aged ≥ 63 years than in those who were < 63 years (*p* = 0.0097 and 0.016 for HMR_E_ and HMR_L_, respectively; Fig. 2). If the lower limit is defined as the 2.5% quantile of 2.3 HMR_L_ for all patients, then 2.5 and 2.0 would be the lower limits in patients with median ages of < 63 and ≥ 63 years, respectively. The lower limit of HMR_E_ was 2.2 for all patients, and 2.4 and 2.1 in patients with age < 63 and ≥ 63 years, respectively. Washout rates calculated using the three formulae did not significantly correlate with age (Fig. 3).

## Discussion

A weak age-dependent decline in HMR was revealed in the JSNM working group ^123^I-MIBG normal databases. Since HMR has been used as a basis for the prognostic evaluation of patients with HF [1, 3, 5, 11] and differential diagnoses of Lewy body diseases [6, 8], this tendency should be considered to appropriately understand clinical results of ^123^I-MIBG uptake. The effect of age notably appeared only after standardization for collimator differences, which showed the importance of correction for collimator types when different studies include various camera-collimator types [18, 19, 21].

Although age was considered as an important factor for the prognosis of HF, the incidence of comorbidities such as diabetes, hypertension ischemic heart disease, and renal dysfunction might also increase with age. Therefore, the tendency of only age versus HMR should be examined in near-normal individuals. The JSNM working group database includes multicenter data, and patients with underlying cardiac disease and those with medications for diabetes, hypertension and neurological diseases were carefully excluded [15, 16]. Therefore, although the patients were not truly as normal as volunteers, the possibility of primary and secondary cardiac diseases was excluded as far as possible in clinical practice.

Few studies have examined HMR in near-normal individuals. As part of the ADMIRE-HF trial, a cohort comprising 94 control individuals with a 10% likelihood of having coronary artery disease according to normal stress myocardial perfusion imaging, stress echocardiography or coronary angiography findings has been investigated [22]. Correlation analyses did not identify a significant relationship between age and planar HMR values, and suggested only a slightly lower HMR for persons aged > 70 years. Another study of 180 patients with HF also found significantly lower early and late HMR compared with younger patients (*p* < 0.05), although values were adjusted for all remaining significant variables [23]. The uptake of ^123^I-MIBG with respect to HMR in a baseline study of 39 patients with cancer before undergoing chemotherapy, found a decrease in ^123^I-MIBG uptake with aging [24]. However, the mean HMR was 1.85 ± 0.29 (range 1.31–2.62) in that study, and much lower than that in the JSNM working group database (HMR_L_ range 2.12–4.52). Although HMR_L_ was significantly lower among older individuals in the JSNM working group database, the average degree of HMR_L_ decline was ~ 0.2 over 30 years (0.07 for 10 years). Therefore, caution might be required to interpret the findings of studies that include patients with a large age range.

Medications taken by elderly patients might have affected HMR. Although a threshold HMR of 1.6 was set in the ADMIRE-HF study, the effects of several medications determined using heart failure medication scores were not significant, whereas the event group had low HMR [25]. Whether the effect of age and medications affect threshold values for the prognostic application of ^123^I-MIBG should be further examined.

The lower limit of HMR 2.1–2.2 has been used to differentiate DLB from AD in a Japanese multicenter study [8]. A lower limit of 2.2 remained valid for general use according to an assessment of HMR distribution in the JSNM working group database. However, in patients aged ≥ 63 years, the HMR_L_ was lower by 0.2–0.3 compared with patients aged < 63 years. Only one 64-year-old female patient had HMR of < 2.2 in this database, and HMR_E_ and HMR_L_ were 2.1 and 2.0, respectively (decay and background-corrected WR = 17%). Whether this difference is critical to differentially diagnose DLB from AD has not been investigated.

Washout rates did not correlate with age. However, a standard method of calculation is desirable to correctly understand the significance of WR. The third calculation formula using early and late HMR has not been applied in Japan, but it is included in European studies [26] and some studies have used this formula when original heart and mediastinal counts were not available. In this calculation, most of the patients (81%) had higher HMR_L_ than HMR_E_, which does not necessarily indicate that the actual WR calculated by the heart count shows negative values. This means that when HMR_L_ is lower than HMR_E_, increased WR or abnormal sympathetic innervation should be suspected.

Immunohistochemical and histochemical analyses have also uncovered age-dependent changes in the human conduction system [27]. Initial sympathetic dominance found in the infant neural supply to the cardiac conduction system in humans is gradually replaced by sympathetic and parasympathetic co-dominance in adulthood, and a reduced density of conduction tissue innervation with aging might also be reflected in ^123^I-MIBG images [28].

One limitation of the present study is that the number of participants was essentially too small to evaluate physiological changes. Early HMR was not significantly affected by age (*p* = 0.076) while late HMR was significant (*p* = 0.028), which might be due to statistical power from the limited number of patients. Although truly normal volunteers were not available, the JSNM working group databases seem to have practical values to visualize age-dependent changes in aged patients using normal ranges. Another limitation was reproducibility of calculating HMR. However, we minimized inter-operator variations using semi-automatic ROI setting software [20, 29]. In this ROI setting algorithm, an operator pointed into the center of the heart, and the following processing was automatically performed. Tomographic studies might be used to integrate whole heart activity, but the present investigation was limited to planar studies.

## Conclusion

An age-dependent decline in HMR, particularly late HMR, was found using JSNM working group normal ^123^I-MIBG databases after collimator standardization. Although the decline in HMR is relatively small, at about 0.2 over 30 years, MIBG results should be interpreted carefully when the study group includes only aged subjects such as in patients with dementia. However, in clinical practice the lower limit of 2.2 and 2.3 for early and late HMR, respectively, can still be used to determine both early and late HMR, and in patients with borderline HMR it would be important to repeat ^123^I-MIBG studies both in cardiology and neurology during their follow-up periods.
